# Treatment of infected placenta accreta in the uterine horn by transabdominal temporary occlusion of internal iliac arteries: A case report and literature review

**DOI:** 10.1097/MD.0000000000034525

**Published:** 2023-08-11

**Authors:** Wenzhi Xu, Zhibao Liu, Qianqian Ren, Chang Dai, Bo Wang, Yangying Peng, Ling Gao

**Affiliations:** a Department of Obstetrics and Gynecology, Sir Run Run Shaw Hospital, School of Medicine, Zhejiang University, Hangzhou, China; b Key Laboratory of Reproductive Dysfunction Management of Zhejiang Province, School of Medicine, Zhejiang University, Hangzhou, China; c Department of Obstetrics and Gynecology, Sir Run Run Shaw Hospital Affiliated to Zhejiang University School of Medicine Alar Hospital, Alar, China; d Department of Radiology, Sir Run Run Shaw Hospital Affiliated to Zhejiang University School of Medicine Alar Hospital, Alar, China; e Department of Ultrasound, Sir Run Run Shaw Hospital Affiliated to Zhejiang University School of Medicine Alar Hospital, Alar, China; f Department of Obstetrics and Gynecology, Taizhou First People’s Hospital, Taizhou, China.

**Keywords:** cornual pregnancy, internal iliac artery occlusion, placenta increta, retained placenta

## Abstract

This case report aims to describe the treatment of infected placenta accreta in the uterine horn by transabdominal temporary occlusion of internal iliac arteries.

**Patient concerns:**

A 29-year-old female patient had a history of retained placenta for 28 days after labor induction in the second trimester of pregnancy because of fetal malformation.

**Diagnoses:**

Placenta accreta in the uterine horn was diagnosed by 3-dimensional ultrasound and magnetic resonance imaging, and the diagnosis was confirmed during the operation.

**Interventions:**

Laparotomy was performed to remove the placenta and repair the uterine defect after temporary occlusion of both internal iliac arteries.

**Outcomes:**

Body temperature and inflammatory markers were elevated at admission but returned to normal on the second day after surgery. Normal menstruation resumed approximately 1 month postoperatively. Ultrasound examination showed that the shape of the uterine cavity was normal. No postoperative complications were observed.

**Lessons:**

Temporary occlusion of the internal iliac artery can help effectively manage infected placenta accreta in the uterine horn.

## 1. Introduction

Abnormally invasive placenta (AIP) is an abnormal trophoblast invasion into the myometrium due to impaired decidua basalis.^[1]^ The incidence of AIP in China has risen to 2.1% over the past 2 decades because of the increased number of cesarean sections.^[2]^ Furthermore, the incidence of AIP increased approximately 60-fold worldwide from the 1960s to 2002 (from 1 in 30,000 pregnancies to 1 in 533 pregnancies).^[3]^

AIP is classified into placenta increta and percreta according to the depth of trophoblast invasion. Abnormal trophoblast invasion can result in massive hemorrhage and serious complications, including disseminated intravascular coagulation, shock, and multiorgan dysfunction.

Internal iliac artery (IIA) occlusion is historically performed as a life-saving strategy to reduce uterine perfusion, especially when technical resources are limited and other conservative treatments have failed.^[4,5]^ The most commonly used approaches to block blood flow to IIA are preoperative balloon occlusion and intraoperative arterial ligation. This study presented the case of a patient with infected placenta accreta in the uterine horn treated by transabdominal temporary occlusion of IIA, thus serving as a reference for AIP treatment.

## 2. Case report

**Figure 1.**
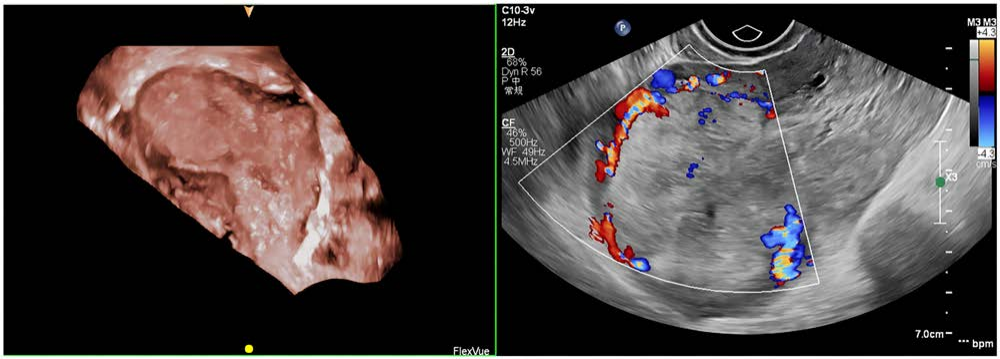
Three-dimensional ultrasound images of uterine horn lesions.

**Figure 2.**
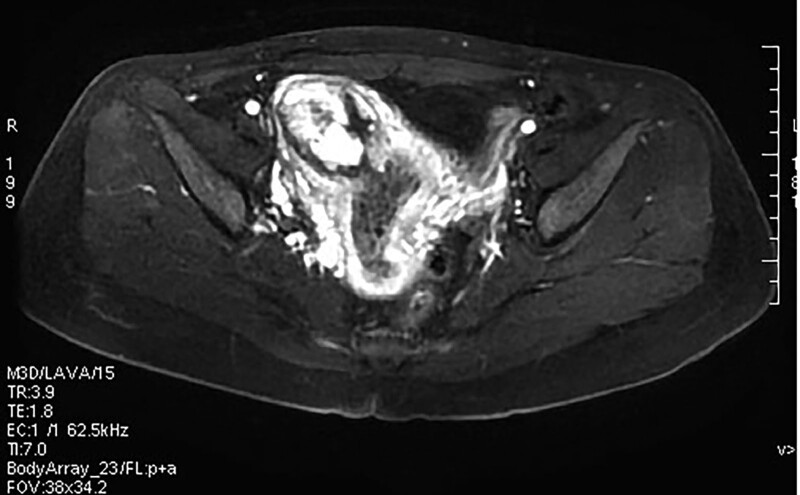
Magnetic resonance image of uterine horn lesions.

**Figure 3.**
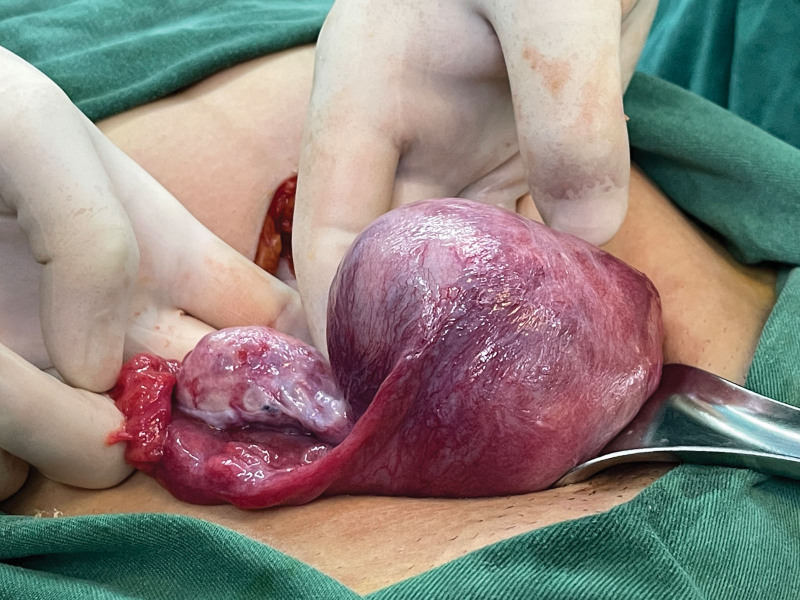
Abundant blood vessels on the serosal surface of the uterus at the lesion.

**Figure 4.**
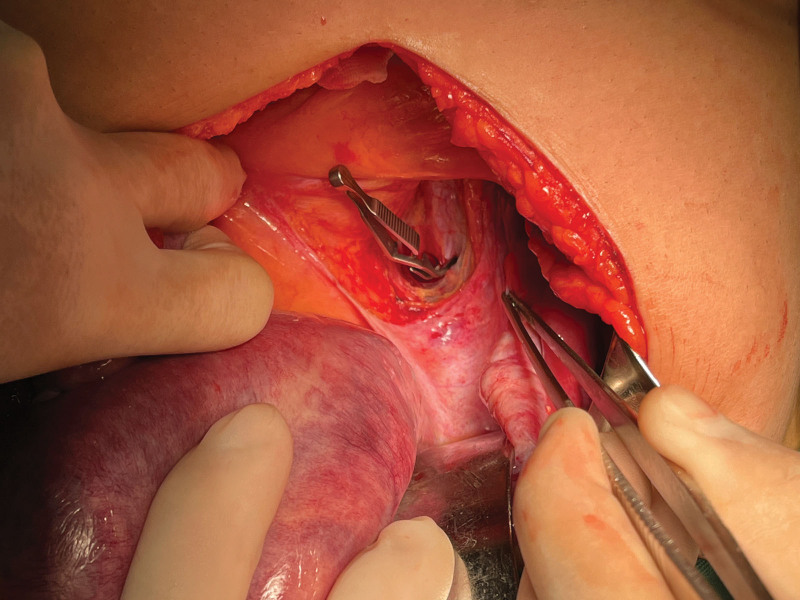
Internal iliac artery occlusion using a laparoscopic bulldog clamp.

**Figure 5.**
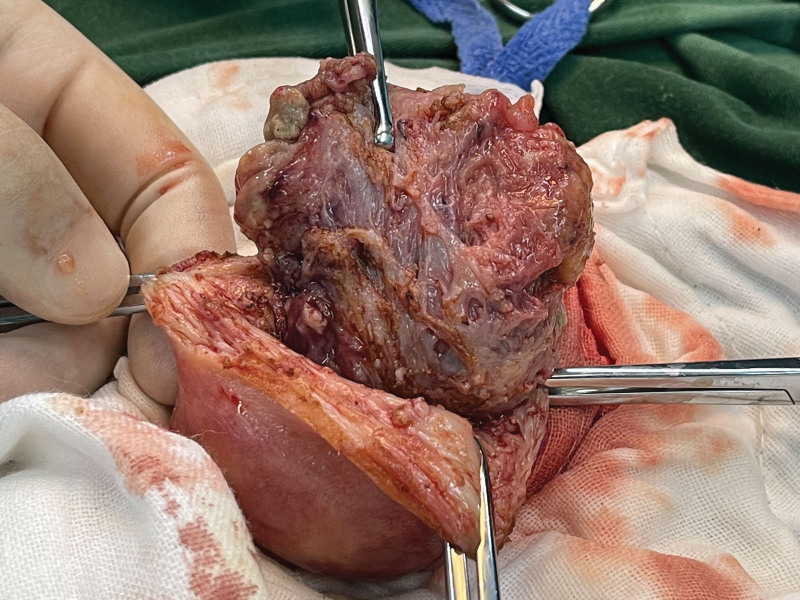
Placenta accreta in the uterine horn.

**Figure 6.**
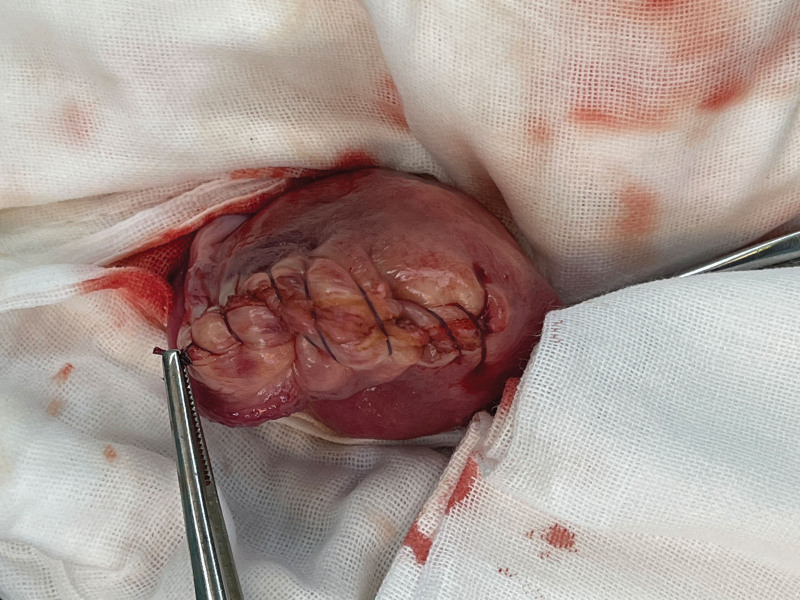
Repaired uterine horns.

**Figure 7.**
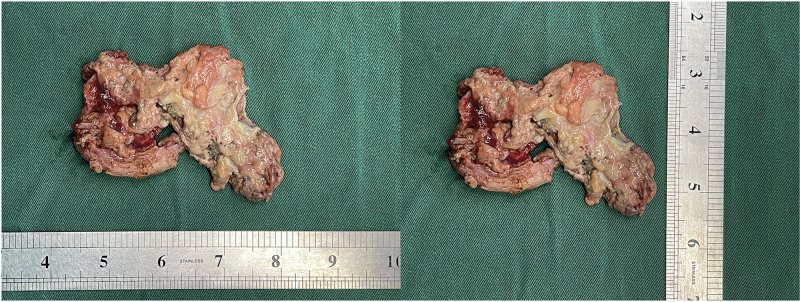
Removed placental tissue specimens.

**Figure 8.**
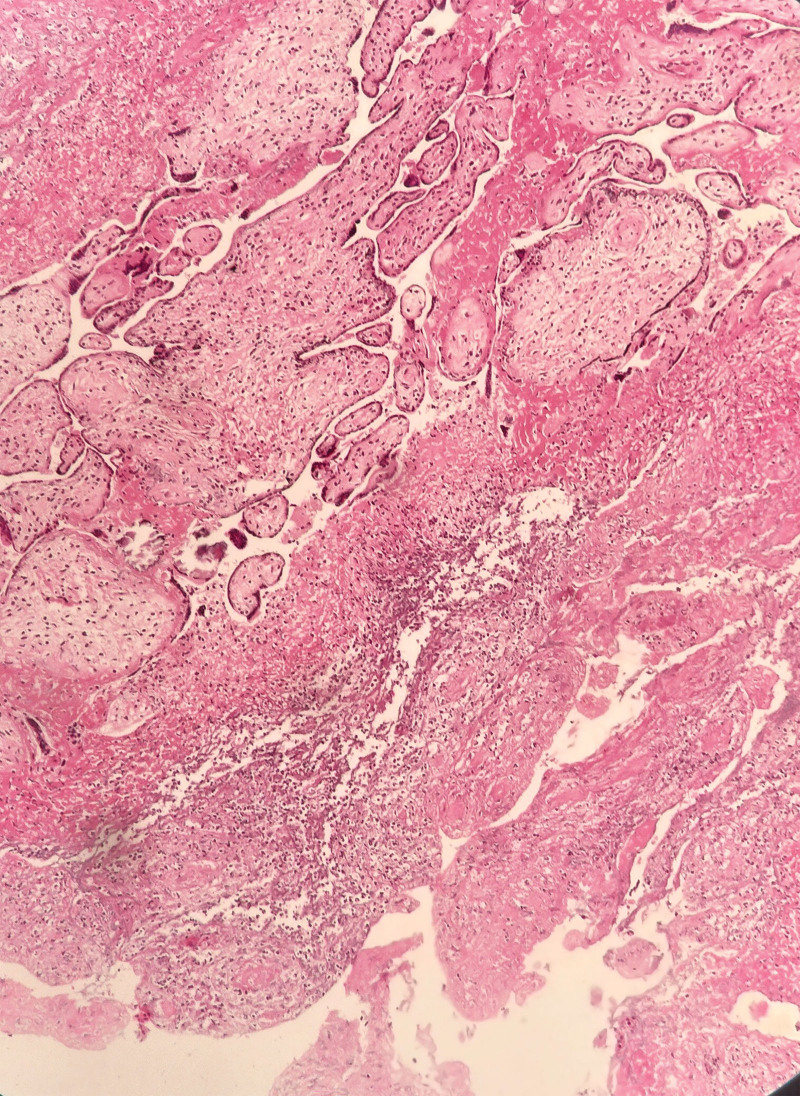
Pathological images of excised placental tissue specimens.

A 29-year-old female patient (gravida 2, para 0) came to our hospital because of retained placenta for 28 days after labor induction in the second trimester of pregnancy. The patient had undergone labor induction at the local hospital at week 22 because of fetal malformation. The placenta was not separated from the uterus during labor. During conservative treatment at the local hospital, the patient experienced lower abdominal pain and fever, and her body temperature reached 39 °C. The patient was a housewife with a history of abortion and no history of psychological stress. Gynecological examination revealed a small amount of discharge from the external os of the cervix, uterine flexion, uterine enlargement, and mild tenderness. Hematological and biochemical tests showed the following findings: hemoglobin concentration, 99 g/L; white blood cell count, 14.68 × 10^9^/L; neutrophil percentage, 84.2%; C-reactive protein, 108.7 mg/L; procalcitonin, 0.55 ng/mL; serum β-human chorionic gonadotropin, 66.56 mIU/mL. Magnetic resonance imaging and 3-dimensional ultrasound showed that the placenta remained in the right uterine horn and invaded the myometrium (Figs. 1 and 2). The preliminary diagnosis was residual placenta combined with infection and placenta accreta in the right uterine horn. Laparotomy and temporary occlusion of both internal iliac arteries were planned to remove the placenta and repair the uterine defect. The patient and her family were fully informed of the surgical procedure and signed informed consent before the operation. A medical team from several departments, including gynecology, urology, imaging, anesthesiology, and blood bank, was convened to improve surgical safety. Under general anesthesia, a 5 cm incision was made between the umbilicus and pubis. The right uterine horn was enlarged and convex, and abundant blood vessels were visible on the surface (Fig. 3). After dissecting the pelvic walls and confirming the position of the ureter, both internal iliac arteries were blocked 3 cm distally from the common iliac artery bifurcation using a removable bulldog clamp (Aesculap, Tuttlingen, Germany) (Fig. 4). The uterine serosa in the right uterine horn was incised longitudinally to allow the visualization of the accreta, and the myometrium invading the placenta was removed (Fig. 5). After clearing the intrauterine fluid, the uterine incision and uterine cavity were washed repeatedly, and the incision was closed with 2-0 absorbable suture using a baseball stitch technique (Fig. 6). Then, the clamp was removed. Total operation time was 45 minutes, the iliac arteries were blocked for 17 minutes, and the volume of intraoperative blood loss was 20 mL. The placental specimen (Fig. 7) was foul-smelling. The patient recovered smoothly after the operation, with minor vaginal bleeding, no fever, no abdominal pain, no iliac wall rupture, no arterial thrombosis, and no pelvic organ ischemia. Moreover, all inflammatory markers returned to normal on the second day after surgery, and the patient was discharged from the hospital. Histopathology examination revealed inflammation and necrosis in the placenta (Fig. 8). The patient resumed menstruation 37 days after the operation, and 3-dimensional ultrasound examination showed that the shape of the uterine cavity was normal.

The patient gave written informed consent for the publication of this case. The institutional review board of our hospital deemed that the study did not require approval by a committee.

## 3. Discussion

Placental remnants can be found after miscarriage, labor induction, transvaginal delivery, and cesarean section. Placenta accreta, including increta and percreta, is one of the leading causes of retained placenta. Placenta accreta is the abnormal attachment of the placenta to the uterine wall, such that chorionic villi invade the myometrium or adjacent organs. This disorder accounts for 23% to 64% of cases receiving peripartum hysterectomy.^[6]^

In our patient, the diagnosis of AIP was confirmed by ultrasonography (with the loss of the retroplacental clear zone, myometrial thinning, and subplacental hypervascularity), magnetic resonance imaging (with thinned or irregular myometrium near the placenta), surgical report, or postoperative pathological results. The retained placenta after labor induction invaded the right uterine horn, and histopathology revealed placental inflammation and necrosis.

According to the characteristics of our patient, 2 aspects of treatment need to be considered: the timing of surgical treatment and how to reduce perioperative bleeding and protect fertility as much as possible. With regard to surgical timing, studies have shown that delayed surgery with a 2.7-month postpartum interval may have similar clinical efficacy and long-term surgical outcomes to those of emergency surgery in the treatment of placental residues.^[7]^ Therefore, these patients can benefit from elective surgery. In this case, the surgical indication was based on the presence of infection or residual necrosis after conservative treatment. As our patient had fertility requirements, the best effort was needed to remove the placental tissue and preserve as much myometrial tissue as possible, which required the effective management of intraoperative bleeding, a clear surgical field, and shorter operation time. Therefore, preventive measures to reduce intraoperative bleeding can improve recovery.

The preoperative placement of IIA catheters with intraoperative endovascular balloon occlusion has become a standard procedure to prevent bleeding. IIA occlusion decreased blood flow to pelvic organs by 49% and reduced pulse pressure by 85% while preventing the complete blockage of blood supply.^[8]^ Moreover, collateral circulation via the obturator, lumbar, sacral, rectal, ovarian, and femoral arteries can prevent pelvic ischemia during balloon occlusion of the IIAs.^[9]^

Prophylactic balloon occlusion of the iliac arteries is an interventional procedure, and its effectiveness in controlling blood loss in placenta accreta remains controversial.^[9,10]^ Balloon occlusion of IIAs is widely performed in high-resource settings to reduce intraoperative blood loss in patients with AIP. However, serious complications may occur, including IIA thrombosis, acute lower extremity ischemia, arterial pseudoaneurysms, and arterial rupture. In contrast, IIA ligation is cheap, easily available, and can be performed in 10 minutes by an experienced surgeon. Instead of isolating and ligating the IIAs using traditional approaches, occlusion was performed in our patient using vascular clamps. These clamps do not significantly affect ovarian blood supply and can reduce the damage to ovarian reserve function.

Notwithstanding these advantages, IIA thrombosis and ischemia-reperfusion injury of pelvic organs may occur because of prolonged intraoperative vascular occlusion, hemodynamic changes, and the hypercoagulable state of blood during pregnancy. Therefore, removing the clamps intermittently during long surgeries is advisable if conditions permit. Furthermore, anticoagulant drugs and compression stockings should be used postoperatively to prevent thrombosis.

We transiently occluded the internal iliac arteries via the transabdominal approach. Compared with traditional interventions, this approach causes less trauma and bleeding and improves recovery. In addition, the effects of occlusion can be observed in real time during the operation, improving the management of bleeding. As a result, our patient recovered well postoperatively with less bleeding, and inflammatory markers returned to normal shortly after surgery.

However, the proposed treatment has limitations. First, the transient occlusion of the bilateral internal iliac arteries requires surgical skill and experience, placing high demands on the surgical team. Surgical competence and experience determine the success of IIA occlusion. Surgeons need to be familiar with the anatomy of the pelvic organs and retroperitoneal vessels, locate and block the IIA promptly, and effectively manage emergencies such as failed occlusion, vessel rupture, retroperitoneal hematoma, and hysterectomy. In addition, little is known about the long-term efficacy and safety of bilateral IIA occlusion.

## 4. Conclusion

This case report highlights the importance of managing placenta accreta by a multidisciplinary team. The temporary occlusion of both internal iliac arteries using removable clamps effectively reduces intraoperative bleeding and facilitates the removal of the placenta. Early diagnosis and timely surgical intervention improve the prognosis and maximally preserve the fertility of the patient. However, more clinical studies and follow-up data are necessary to evaluate the long-term efficacy and safety of this approach.

## Acknowledgements

We acknowledge TopEdit (www.topeditsci.com) for the linguistic editing and proofreading during the preparation of this manuscript.

## Author contributions

**Data curation:** Zhibao Liu, Qianqian Ren, Chang Dai, Bo Wang, Yangying Peng.

**Writing – review & editing:** Wenzhi Xu, Ling Gao.
